# Spatiotemporal Characteristics of Bacillary Dysentery from 2005 to 2017 in Zhejiang Province, China

**DOI:** 10.3390/ijerph15091826

**Published:** 2018-08-24

**Authors:** Congcong Yan, Yijuan Chen, Ziping Miao, Shuwen Qin, Hua Gu, Jian Cai

**Affiliations:** 1 Department of Preventive Medicine, School of Medicine, Ningbo University, Ningbo 315200, China; kb981147121@163.com; 2 Zhejiang Provincial Center for Disease Control and Prevention, Hangzhou 310051, China; yjchen@cdc.zj.cn (Y.C.); zpmiao@cdc.zj.cn (Z.M.); swqin@cdc.zj.cn (S.Q.); 3 Zhejiang Provincial Center for Medical Science Technology & Education, Hangzhou 310006, China

**Keywords:** bacillary dysentery, epidemiological characteristics, spatiotemporal characteristics, high-risk areas

## Abstract

Background: This study aimed to analyze the epidemiological and spatiotemporal characteristics of bacillary dysentery in Zhejiang Province and to provide the basis for its monitoring, prevention and control. Methods: This study included cases registered in China Information System for Diseases Control and Prevention from 1 January 2005 to 31 December 2017 in Zhejiang. Descriptive methods were employed to investigate the long trend of this disease: gender distribution, high-risk population, seasonality, and circular distribution was explored to detect the peak period; incidence maps were made to show the incidence trend of disease at county level; spatial autocorrelation was explored and the regions with autocorrelation were detected; and spatiotemporal scan was conducted to map out the high-risk regions of disease and how long they lasted. Statistical significance was assumed at *p* value of <0.05. Results: A total of 105,577 cases of bacillary dysentery were included, the incidence declining sharply from 45.84/100,000 to 3.44/100,000 with an obvious seasonal peak from July to October. Males were more predisposed to the infection than females. Pre-education children had the highest proportion among all occupation categories. Incidence in all age groups were negatively correlated with the year (*p* < 0.001), and the incidences were negatively correlated with the age groups in 2005–2008 (*p* = 0.022, 0.025, 0.044, and 0.047, respectively). Local autocorrelation showed that counties in Hangzhou were high-risk regions of bacillary dysentery. The spatiotemporal scan indicated that all clusters occurred before 2011, and the most likely cluster for disease was found in Hangzhou, Jiaxing and Huzhou. Conclusions: The incidence of bacillary dysentery in Zhejiang from 2005 to 2017 featured spatiotemporal clustering, and remained high in some areas and among the young population. Findings in this study serve as a panorama of bacillary dysentery in Zhejiang and provide useful information for better interventions and public health planning.

## 1. Background

Bacillary dysentery, a common infectious disease, is caused by the bacteria of the genus *Shigella* and is usually transmitted by fecal–oral route or sometimes contact through flies, food and water [1,2]. People with poor hygiene habits or in poor sanitation and with a lack of sanitary water supply are more susceptible to infection [3,4,5,6,7]. People of all ages can get infected with bacillary dysentery, and infection rates are higher among pre-school children and young adults [1]. Its incubation period is generally 1–4 days. Most patients can be cured in 1–2 weeks, but with short immunity, and the infected are susceptible to multiple infections due to the absence of cross-protection [1]. *Shigella* has a couple of serotypes, classified as *Shigella dysenteriae*, *Shigella flexneri*, *Shigella boydii* and *Shigella sonnei* according to their biochemical reactions and O antigen. Their epidemiological patterns are different, with *Shigella flexneri* mainly prevailing in developing countries and *Shigella sonnei* mainly in developed countries [8,9]. With socio-economic development, *Shigella sonnei* has gradually replaced *Shigella flexneri* as the major cause of epidemics in the new industrial parks in Thailand, Iran, South Korea, Taiwan, Vietnam, China and Bangladesh [10,11,12,13,14,15,16]. *Shigella* episodes are a global public health issue with an estimated 165 million cases around the world and more than 100 million occurring in developing countries [17]. Although the incidence of bacillary dysentery in the world has declined significantly in recent years, it remains high in developing countries [8,10,18,19]. The incidences in China remains high with an estimate of 15.29 per 100,000 population in 2012 and 11.24 per 100,000 population in 2014 [20,21]. The incidence of bacillary dysentery differs in each region; for example, the average annual incidences were 22.12 per 100,000 population from 2004 to 2014 in Sichuan Province, 24.48 per 100,000 population in Baise from 2004 to 2012, and from 6.00 to 15.80 per 100,000 population during 2010–2015 in Hunan Province [22,23,24]. Few studies have been done about this disease in Zhejiang Province, resulting in knowledge gaps in the aspects of high-risk population, high-risk regions, peak period and spatiotemporal characteristics. Geographical information system (GIS) has been widely used to analyze the spatiotemporal characteristics of diseases, helping to monitor and prevent the communicable diseases [25]. For example, it is easy for people to view the incidence difference of bacillary dysentery between regions via an incidence level map [26,27]. Global autocorrelation is employed to detect the clusters of a disease, local autocorrelation is employed to examine regional patterns and ascertain the exact clustering location, and Moran’s I can be used to fulfill such purposes [28,29,30]. Spatiotemporal scan is employed to detect diseases in time and space, verify random distribution of the disease in time and space, and ascertain the number of cases in a region, the scope of the disease and other information indicating possible areas of high risk [31]. As it involves demographic information, spatiotemporal scan is more useful than other methods in the detection of clustering in low-incidence areas [32]. Special clusters are usually identified by comparing the observed number with the expected one using Log-likelihood Ratio (*LLR*) [33].

In this study, we intended to conduct a full scope analysis of the epidemiological characteristics of bacillary dysentery and identify the high-risk regions, both spatially and temporally, using above-mentioned methods, thus providing the basis for the monitoring, prevention and control of bacterial dysentery in Zhejiang Province.

## 2. Materials and Methods

### 2.1. Study Areas

**Figure 1:**
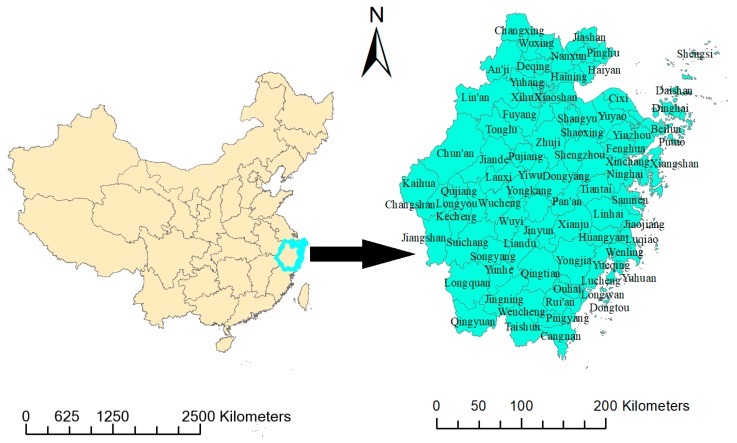
Administrative division map in Zhejiang at county level.

Zhejiang, located in southeast China (27°12′–31°31′ North, 118°–123° East) with a subtropical monsoon climate, governs 11 municipalities and 90 counties with a population of 55,000,000 at the end of 2017 (Figure 1).

### 2.2. Data Collection

This study covered the whole population in Zhejiang Province, and included all cases registered in China Information System for Diseases Control and Prevention and diagnosed as bacillary dysentery by clinical doctors in Zhejiang Province from 2005 to 2017 in accordance to the unified diagnostic criteria promulgated by Ministry of Health of the People’s Republic of China [34]. 

Demographic data were retrieved from China Information System for Diseases Control and Prevention.

### 2.3. Statistical Software

In this study, ArcGIS software (version 10.1, ESRI Inc.; Redlands, CA, USA) was used for mapping and autocorrelation analysis. The spatiotemporal clusters were detected with SatScan (version 9.4. Martin Kulldorff, National Cancer Institute, Bethesda, MD, USA; Farzad Mostashari, New York City Department of Health and Mental Hygiene, NY, USA). SPSS (version 16.0, IBM Inc., Chicago, IL, USA) was employed for Spearman’s rank correlation, Wilcoxon rank sum test. All results were considered statistically significant if *p* < 0.05 for both sides.

#### 2.3.1. Circular Distribution

Seasonality of bacillary dysentery incidence in Zhejiang Province was characterized by analyzing peak month and peak days. Peak days are the day with the most cases of the disease in the whole year. Circular distribution is generally applicable to the seasonal disease with only one peak period [35]. To calculate circular distribution, 365 days in a year is changed to 360°, and one day is represented by 0.9863°. The monthly median is taken as the middle value of the group and is then converted into degrees. Thus, circular distribution is calculated as the following [36], where *f_i_* is the monthly cases of disease, *α_i_* is monthly degree, *n* is the yearly cases of disease, *r*-value is the index for degree of dispersion, and *s* is standard deviation of the angle.

(1) $$r=\sqrt{{\left[\left(\sum f_{i}\cos\alpha_{i}\right)/n\right]}^{2}+{\left[\left(\sum f_{i}\sin\alpha_{i}\right)/n\right]}^{2}}\;$$

(2) $$s=\frac{180{}^{\circ}}{\pi }\sqrt{-2\ln r}\;$$

#### 2.3.2. Incidence Maps and Spatial Autocorrelation

The incidence map was made to show the generally distribution of bacillary dysentery at county level. Spatial autocorrelation analysis helps to map the spatial connections in adjacent geographic units, indicating the extent of uneven distribution of values [37]. It can be used to identify various spatial clusters based on spatial weight matrices at a point time [38]. In this study, global and local Moran’s I, widely used in studies of infectious disease, were employed to estimate the spatial autocorrelation [39,40,41]. Global Moran’s I was used to explore the spatial autocorrelation in the whole province, and local Moran’s I to explore the spatial autocorrelation. Global as well as local Moran’s I is between −1 and 1. A value approaching 1 indicates a positive correlation, and approaching −1 a negative correlation. Clusters were divided into high–high (HH, high incidence surrounded by high incidence), high–low (HL, high incidence surrounded by low incidence), low–high (LH, low incidence surrounded by high incidence), and low–low (LL, low incidence surrounded by low incidence) [42].

#### 2.3.3. Spatiotemporal Scan

Spatiotemporal scan analysis was performed to identify the most likely clusters (with the largest *LLR* values), auxiliary clusters (with statistically significant *LLR*s), and clustering time [43]. In this study, we collected data of the permanent residents, the latitude and longitude of each county and organized the case data by month at the county level. When the high-incidence clusters were calculated, retrospective space-time analysis was performed, Poisson model was chosen as probability model to estimate high-incidence areas, and the maximum spatial cluster size was defined as a circle with a 60-kilometer radius.

## 3. Results 

### 3.1. Epidemiological Trends

**Figure 2:**
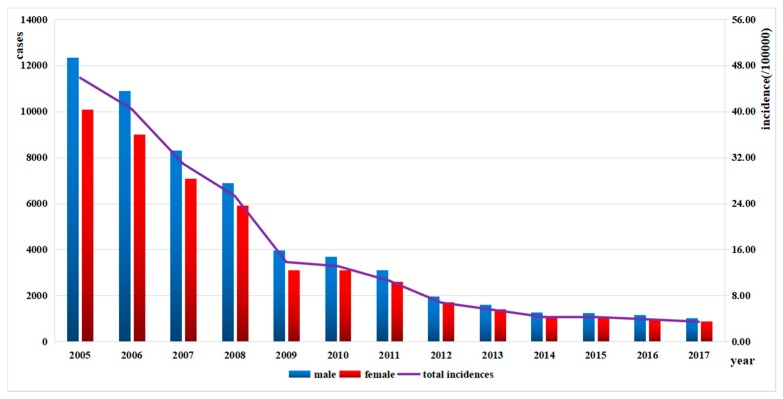
Total incidences and gender distribution of bacillary dysentery in Zhejiang Province from 2005 to 2017.

**Figure 3:**
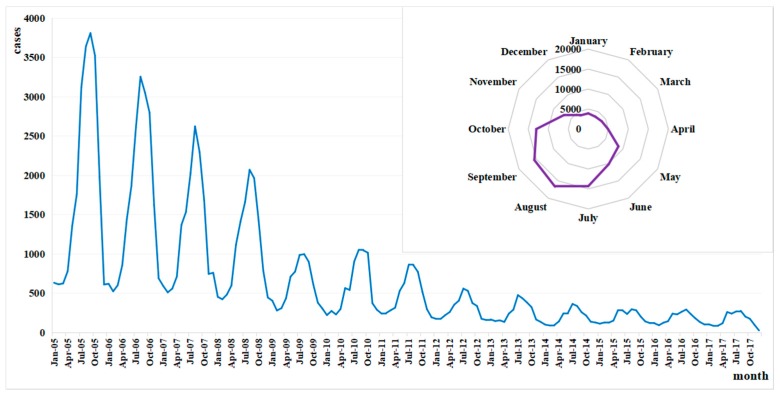
Monthly distribution of bacillary dysentery in Zhejiang Province from 2005 to 2017.

**Figure 4:**
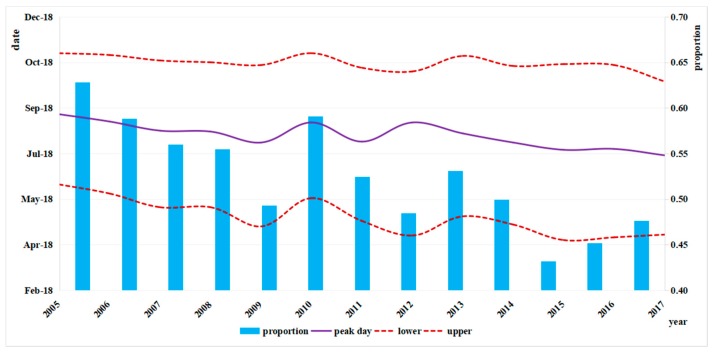
Peak days of bacillary dysentery and proportion from July to October in Zhejiang Province in 2005–2017.

Exclusion of those with unclear addresses (less than 1%) left a total of 105,577 patients diagnosed as bacillary dysentery in Zhejiang Province from 2005 to 2017, with an obvious decline in the incidence from 45.84/100,000 in 2005 to 3.44/100,000 in 2017 (Figure 2). The annual average rate of decline was 6.84 per 100,000 population, but it decreased by 11.50 per 100,000 population in 2009. More than half of the infections occurred in July, August, September and October. Seasonality was obvious with a peak in summer and autumn from July to October during 2005–2017 (Figure 3). The peak days of infections always fell in summer and came earlier every year from August in 2005 to July in 2017. The proportion of cases from July to October showed a declining trend (Figure 4).

**Figure 5:**
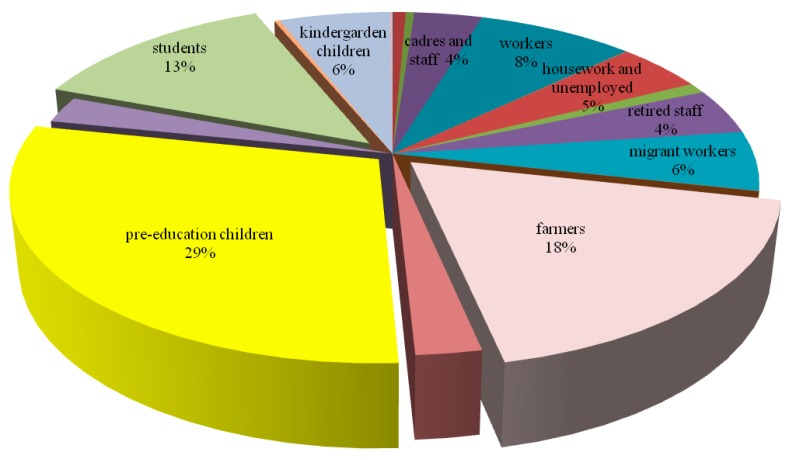
Occupation distribution of bacillary dysentery in Zhejiang Province from 2005 to 2017.

Gender distribution showed that the number of cases in males was higher than in females every year (Figure 2). The incidences in all age groups declined from 2005, and the incidences and the number of cases in 0–5-year group were much larger than other age groups. The Spearman’s rank correlation showed that the incidences in every age group were negatively correlated to year (*p* < 0.001), while the incidences were negatively correlated to age groups only during 2005–2008 (*p* = 0.022, 0.025, 0.044 and 0.047, respectively) (Table 1). In terms of occupation, the proportions of pre-education children, farmers and students were consistently higher than others every year, and the differences between occupations were statistically significant (*p* < 0.001) (Figure 5).

### 3.2. Incidence Maps

**Figure 6:**
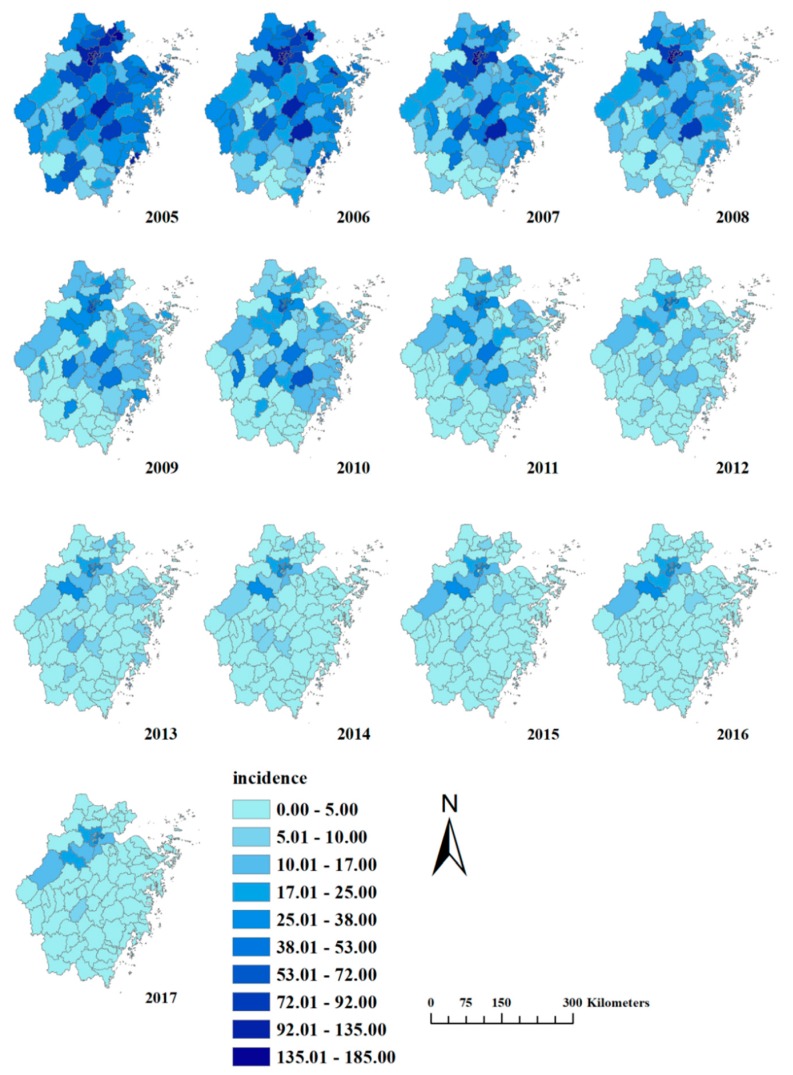
Incidence maps of bacillary dysentery in Zhejiang Province from 2005 to 2017.

The incidences of bacillary dysentery were mapped at county level in Zhejiang Province from 2005 to 2017 (Figure 6), and the maps indicated that counties in and around Hangzhou had higher incidences than other places. Counties in Hangzhou being excluded, the counties with high incidences were found to be scattered. The number of counties with an incidence over 10.00/100,000 declined from 78 in 2005 to 10 in 2017. 

### 3.3. Autocorrelation Analysis and Spatial Stratified Heterogeneity

**Figure 7:**
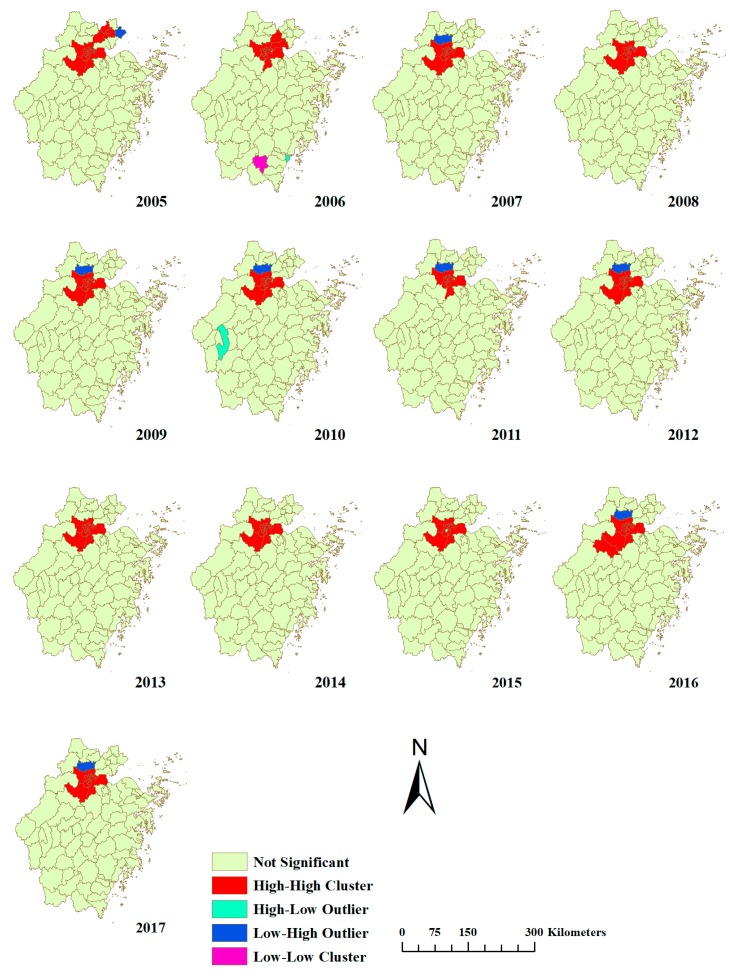
Local autocorrelation of bacillary dysentery in Zhejiang Province from 2005 to 2017.

All the values of Moran’s I for global autocorrelation were positive (0.522, 0.520, 0.638, 0.721, 0.556, 0.510, 0.693, 0.690, 0.677, 0.704, 0.723, 0.690 and 0.739, from 2005 to 2017, respectively) (*p* < 0.001 each year), suggesting a clustering distribution at the provincial level every year (Table 2). Local autocorrelation detected 128 high–high, 10 low–high, 2 high–low and 1 low–low clusters (Figure 7). High–high clusters were observed in Hangzhou every year from 2005 to 2017.

### 3.4. Spatiotemporal Cluster Analysis

**Figure 8:**
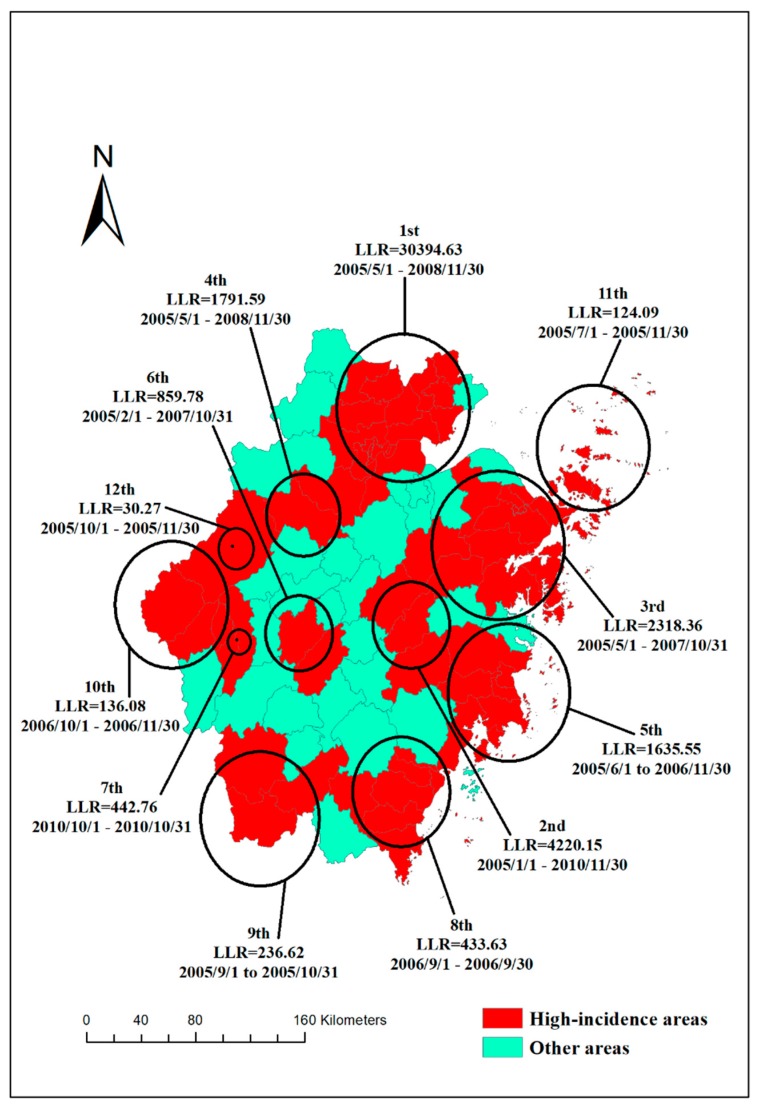
High rates clusters of bacillary dysentery in Zhejiang Province from 2005 to 2017.

Spatiotemporal cluster analysis showed 12 high-incidence clusters of bacillary dysentery in Zhejiang Province from 2005 to 2017. High-incidence clusters were detected in 64 counties, with all clusters occurring before 2011. The most likely clusters were found from May 2005 to November 2008 (*LLR* = 30394.63, *p* < 0.001) in 18 counties (mainly in Hangzhou, Jiaxing and Huzhou). Other clusters (the third most likely, the fifth most likely, the eighth most likely, and the eleventh most likely) were observed in the coastal regions (Ningbo, Taizhou, Wenzhou and Zhoushan). In all clusters, the longest duration was 43 months and the shortest only one month (Figure 8).

## 4. Discussion

In this study, we filled some gaps in knowledge of bacillary dysentery in the aspects of its long-term trend, high-risk population, peak period and comparative spatiotemporal clustering, which provided solid evidence for targeted strategy in the prevention and control of bacterial dysentery in Zhejiang. The average incidence of bacillary dysentery declined by a large margin since 2005 to a low level (4.25 per 100,000 population) in 2014, which was lower than the average in China as a whole and other Chinese provinces [21,44,45] and lower than that reported in the United States (6.5 per 100,000 population) [46]. Such findings suggest that prevention and control have been effective. Zhejiang Province began to manage water supply and built sanitary toilets on a large scale in 2008, and these measures could have significantly reduced the incidences of infectious diseases transmitted by fecal–oral route including bacillary dysentery [47,48,49]. Some studies showed that the incidences of the infections featured seasonality with an obvious temporal peak appearing from July to October (in summer and autumn) each year, which was consistent with our results [50,51,52]. Zhejiang, a coastal province located in southeast China, enjoys a subtropical monsoon climate with high temperatures and abundant precipitation in summer, accounting for the high incidences of bacillary dysentery. Preference for cold or uncooked food in Zhejiang could be another reason for the high incidences in summer [53,54]. Declined cases from July to October meant less obvious seasonality from 2009, and the risk factors for disease also decreased at the same time, suggesting that measures including water management and hygiene improvement adopted by the government from 2008 might have made a huge contribution.

The incidences of bacillary dysentery declined by a large margin in both men and women and the incidences in the former were always statistically significantly higher than those in the latter. Men were more likely to be infected because they performed more outdoor activities, drank raw water and had poorer hygiene habits after work [55,56,57]. Consistent with other studies [21,58,59], among the pre-education children, the largest group of bacillary dysentery, 0–5-year-old group still had the highest incidences, although all incidences declined greatly, warranting strengthened monitoring and interventions for them, e.g. teaching good hygiene practices. The number of counties with incidences higher than 10.00/100,000 decreased, suggesting that the public health measures were effective in recent years. The incidence maps showed that counties in Hangzhou always had the highest incidences across the province from 2005, an observation consistent with the findings of spatiotemporal cluster scan analysis. As the provincial capital, Hangzhou enjoyed rapid economic development, dense population and many immigrants, which may have led to the high incidences of disease [60,61].

Moran’s I was chosen to estimate spatial autocorrelation because it is not easily affected by skewed distribution [62]. At the same time, some studies have shown that Moran’s I focuses more on the co-correlation between spatial objects, and Moran’s I is more widely used [63]. The high–high clusters reflected a high impact of the high-incidence areas on surrounding area. Global autocorrelation and local autocorrelation indicated that the incidence of bacillary dysentery was not randomly distributed at county level in Zhejiang Province, and that there was a spatial correlation every year. Local autocorrelation found constant high–high clusters in Hangzhou with small fluctuations with time, implying more attention should be paid to monitoring and preventing the disease in these areas. 

Spatiotemporal scan reflected clustering. In this study, clusters occurred almost every year from 2005 to 2008. A project aimed at reconstructing the water supply and lavatories was launched in Zhejiang Province in the 1980s, which was further strengthened in 2008 [49]. Given that sanitary interventions effectively reduce the intestinal infectious diseases, the project might have resulted in a sharp decline of bacillary dysentery after 2008. Social and economic development might account for the observation that all high-incidence clusters of bacillary dysentery occurred before 2011. 

## 5. Conclusions

The incidence of bacillary dysentery declined in Zhejiang Province from 2005 to 2017, with high incidences in some areas and peaks in summer and autumn. The findings provide evidence of effective early interventions such as protection and health education of high-risk populations, especially males and children <5 years old; surveillance of the high-risk areas, especially in peak period every year; the management of water resources; and construction of sanitary toilets.

## Figures and Tables

**Table 1:** Correlation between incidences between age groups and year and between incidences every year and age groups.

Age Groups	*r* (Incidence and Year)	*p*	Year	*r* (Incidence and Age Group)	*p*
0–5	−0.928	<0.001	2005	−0.535	0.022
5–10	−0.887	<0.001	2006	−0.527	0.025
10–15	−0.87	<0.001	2007	−0.480	0.044
15–20	−0.866	<0.001	2008	−0.473	0.047
20–25	−0.907	<0.001	2009	−0.421	0.082
25–30	−0.896	<0.001	2010	−0.438	0.069
30–35	−0.882	<0.001	2011	−0.404	0.096
35–40	−0.904	<0.001	2012	−0.367	0.134
40–45	−0.913	<0.001	2013	−0.362	0.14
45–50	−0.929	<0.001	2014	−0.376	0.124
50–55	−0.918	<0.001	2015	−0.352	0.152
55–60	−0.942	<0.001	2016	−0.391	0.109
60–65	−0.94	<0.001	2017	−0.421	0.082
65–70	−0.884	<0.001			
70–75	−0.912	<0.001			
75–80	−0.924	<0.001			
80–85	−0.926	<0.001			
85–	−0.969	<0.001			

**Table 2:** Global autocorrelation of bacillary dysentery in Zhejiang Province from 2005 to 2017.

Year	Moran’s I	Z Score	*p*-Value	
2005	0.522	7.385	<0.001	clustered
2006	0.520	7.352	<0.001	clustered
2007	0.638	9.080	<0.001	clustered
2008	0.721	10.359	<0.001	clustered
2009	0.556	7.864	<0.001	clustered
2010	0.510	7.224	<0.001	clustered
2011	0.693	9.821	<0.001	clustered
2012	0.690	9.844	<0.001	clustered
2013	0.677	9.657	<0.001	clustered
2014	0.704	10.060	<0.001	clustered
2015	0.723	10.277	<0.001	clustered
2016	0.690	9.784	<0.001	clustered
2017	0.739	10.647	<0.001	clustered

## Availability of Data and Materials

The data generated during the study are not publicly available due to regulations, but they can available from the corresponding author through request.
